# Does Sulfoquinovosyl Diacylglycerol Synthase OsSQD1 Affect the Composition of Lipids in Rice Phosphate-Deprived Root?

**DOI:** 10.3390/ijms24010114

**Published:** 2022-12-21

**Authors:** Yafei Sun, Qin Qin, Ke Song, Lijuan Sun, Tingting Jiang, Shiyan Yang, Zhouwen Li, Guohua Xu, Shubin Sun, Yong Xue

**Affiliations:** 1 Institute of Eco-Environment and Plant Protection, Shanghai Academy of Agricultural Sciences, Shanghai 201403, China; 2 Shanghai Key Laboratory of Protected Horticultural Technology, Shanghai Academy of Agricultural Sciences, Shanghai 201403, China; 3 Key Laboratory of Low-Carbon Green Agriculture, Ministry of Agriculture and Rural Affairs, Shanghai 201403, China; 4 Key Laboratory of Plant Nutrition and Fertilization in Low-Middle Reaches of the Yangtze River, Ministry of Agriculture, Nanjing Agricultural University, Nanjing 210095, China

**Keywords:** *OsSQD1*, glycolipids, phospholipids, phosphate, root

## Abstract

Lipids are the essential components of the cell intracellular and plasma membranes. Sulfoquinovosyldiacylglycerol (SQDG) is a glycolipid; glycolipids can replace phospholipids in maintaining phosphate (Pi) homeostasis in plants which are undergoing Pi starvation. Sulfoquinovosyl diacylglycerol synthase 1 (OsSQD1) is a critical enzyme in the first step of catalyzation in the formation of SQDG in rice. In this study, the expression pattern of different zones in roots of *OsSQD1* in response to different Pi conditions is examined, and it is found that *OsSQD1* is highly expressed in lateral roots under Pi-sufficient and -deficient conditions. The root phenotype observation of different *OsSQD1* transgenic lines suggests that the knockout/down of *OsSQD1* inhibits the formation and growth of lateral roots under different Pi conditions. Additionally, the lipid concentrations in *OsSQD1* transgenic line roots indicate that *OsSQD1* knockout/down decreases the concentration of phospholipids and glycolipids in Pi-starved roots. The *OsSQD1* mutation also changes the composition of different lipid species with different acyl chain lengths, mainly under Pi-deprived conditions. The relative transcript expression of genes relating to glycolipid synthesis and phospholipid degradation is estimated to help study the mechanism by which *OsSQD1* exerts an influence on the alteration of lipid composition and concentration in Pi-starved roots. Moreover, in Pi-starved roots, the knockout of *OsSQD1* decreases the unsaturated fatty acid content of phospholipids and glycolipids. To summarize, the present study demonstrates that *OsSQD1* plays a key role in the maintenance of phospholipid and glycolipid composition in Pi-deprived rice roots, which may influence root growth and development under Pi-deprived conditions.

## 1. Introduction

Phosphorus (P) is the most important macronutrient for the growth and development of all organisms and a component of key compounds. For example, phosphorus is essential for DNA, RNA, glycophosphate, intermediates of glycolysis, respiration and photosynthesis, phospholipids (which are used for forming membranes), and various phosphorylated compounds used in a great deal of different reactions [1]. Plants absorb phosphate (Pi) as their main P source. However, Pi usually complexes with metal ions in soil, which makes it hard for plants to take up. In general, the soil Pi concentration may be much lower than that in plant tissues (5–20 mM), resulting in Pi deprivation in plants [1,2]. Consequently, plants have evolved complex mechanisms to cope with starvation, including biochemical and metabolic adaptation, to augment the availability of external and internal Pi [3]. The modification of root architecture, increased secretion of organic acids, and changes in plant mechanisms can help increase Pi uptake capacity [4,5].

Lipids are the major components of prokaryotic and eukaryotic membranes [6]. As well as being the structural components of the plasma and cell intracellular membrane, they also perform a variety functions in signal transduction, stress response, and carbon storage, as well as biological functions in terms of being a source of energy [7]. Plant cells contain many different types of lipids, such as glycolipids, sterol lipids, phospholipids, waxes, sphingolipids, and fatty acids. Phospholipids mainly include phosphatidylglycerol (PG), phosphatidic acid (PA), phosphatidylinositol (PI), phosphatidylcholine (PC), phosphatidylserine (PS), and phosphatidylethanolamine (PE) in higher plants [8,9,10,11]. They contain two fatty acids esterified to the *sn*-1 and *sn*-2 positions of the glycerol backbone and a polar headgroup attached to the *sn*-3 position [12]. Glycolipids, such as monogalactosyldiacylglycerol (MGDG), digalactosyldiacylglycerol (DGDG), and sulfoquinovosyldiacylglycerol (SQDG), have glycerol backbones with two fatty acid molecules at the *sn*-1 and *sn*-2 positions, and have either a phospholipid or a sugar molecule at the *sn*-3 position. Under conditions of Pi starvation, membrane phospholipids can be replaced by nonphosphorus glycerolipids to release more Pi catalyzed by phospholipases [13,14]. This transformation in lipid metabolism occurs in many species of bacteria, Arabidopsis (*Arabidopsis thaliana*), and rice (*Oryza sativa* L.) [15,16,17,18,19,20,21,22,23]. The decrease in phospholipids under conditions of Pi deficiency and their replacement with glycolipids were first discovered in the nonphotosynthetic bacterium *Pseudomonas diminuta* [15]. Subsequently, this mechanism was also found in the photosynthetic purple bacterium *Rhodobacter sphaeroides,* the cyanobacterium *Synechococcus* sp. PCC7942, *Chlamydomonas reinhardtii*, *Oleaginous microalga* and *Nannochloropsis oceanica* [16,17,21,22]. For higher plants, previous studies have shown that Pi deficiency strongly reduces the relative contents of PE, PC, and PG, and induces DGDG and SQDG contents in Arabidopsis shoots [18,19,20]. During leaf development in *Hakea prostrata1* under Pi starvation, mature leaf blades showed delayed greening and decreased chloroplast size or quantity, and extensive lipid remodeling in the course of development by the up- and down-regulation of PG and nonphosphorus glycolipids [24]. Pi-starved rice increased the SQDG, DGDG, and MGDG contents to replace the decreasing PG, PC, PE and PS in shoots [23]. This mechanism leads to the maintenance of fundamental development processes upon Pi deficiency [25,26].

Under Pi-deprivation, a reaction involving nonspecific phospholipase C (NPC4 and NPC5) and another reaction involving phospholipase D (PLDζ1 and PLDζ2), together with PA phosphohydrolase (PAH1 and PAH2) pathways are considered, primarily in order to dephosphorylate phospholipids [13,27,28,29,30,31,32,33]. Plant glycolipid biosynthesis generally begins with the synthesis of fatty acids in plastids. The 16:0 and 18:1-acyl carrier protein product may enter the prokaryotic lipid biosynthesis pathway in the plastids, and also release fatty acids and enter the eukaryotic pathway [34]. DGDG is the main substitute for the decreased phospholipids in leaves and roots [30]. In addition, SQDG also replaces PG in the shoots of Arabidopsis and rice [18,23,35]. In Arabidopsis, Pi deficiency induced the relative expression of MGDG synthase (MGD2/MGD3) [36,37,38], DGDG synthase (DGD1/DGD2) [39,40], UDP-glucose pyrophosphorylase 3 (UGP3) [41] and sulfoquinovosyl dacylglycerol synthase 1 and 2 (SQD1/SQD2) [42,43]. As shown in previous studies, SQD1 is a member of the short-chain dehydrogenase/reductase (SDR) enzyme superfamily [44]. The crystal structure of SQD1 was identified in Arabidopsis [45]. It embodies a tightly bound NAD+ cofactor functioning as a hydride acceptor and is structurally analogue to other sugar nucleotide modifying enzymes [45,46,47]. In Arabidopsis, lipidomic analysis revealed significant inductions in 1,2-diacyl-3-O-α-glucuronosylglycerol (GlcADG), PI, and PG and reductions in SQDG and PE in the *SQD1* mutant, contrasting with the wild type. However, there was no significant difference in the phenotypes of the *sqd1* mutant and the wild type upon Pi limitation [19,41]. In rice, *OsSQD1* was reported to be the gene encoding UDP-sulfoquinovose synthase, which plays important roles in the development of various tissues. Furthermore, *OsSQD1* plays a role in maintaining the homeostasis of P and sulfur in shoots. The knockdown of *OsSQD1* significantly reduces the content of glycolipids and induces phospholipids in shoots under conditions of sufficient and deficient Pi [23]. However, its effect on lipid metabolism in roots is unclear.

In this study, we identified the expression pattern of *OsSQD1* in roots and analyzed the root phenotype of knockout/down lines under -P conditions. Knockout/downregulation of *OsSQD1* inhibited the formation and development of lateral roots, especially under −P conditions. Moreover, lipid metabolism analysis revealed that mutation of *OsSQD1* decreased the concentration and unsaturated double bonds of both phospholipids and glycolipids in Pi-deprived roots. Fatty acids with different acyl chain lengths were also triggered by *OsSQD1* mutation.

## 2. Results

### 2.1. OsSQD1 Plays a Function in Formation and Development of Lateral Root

**Figure 1:**
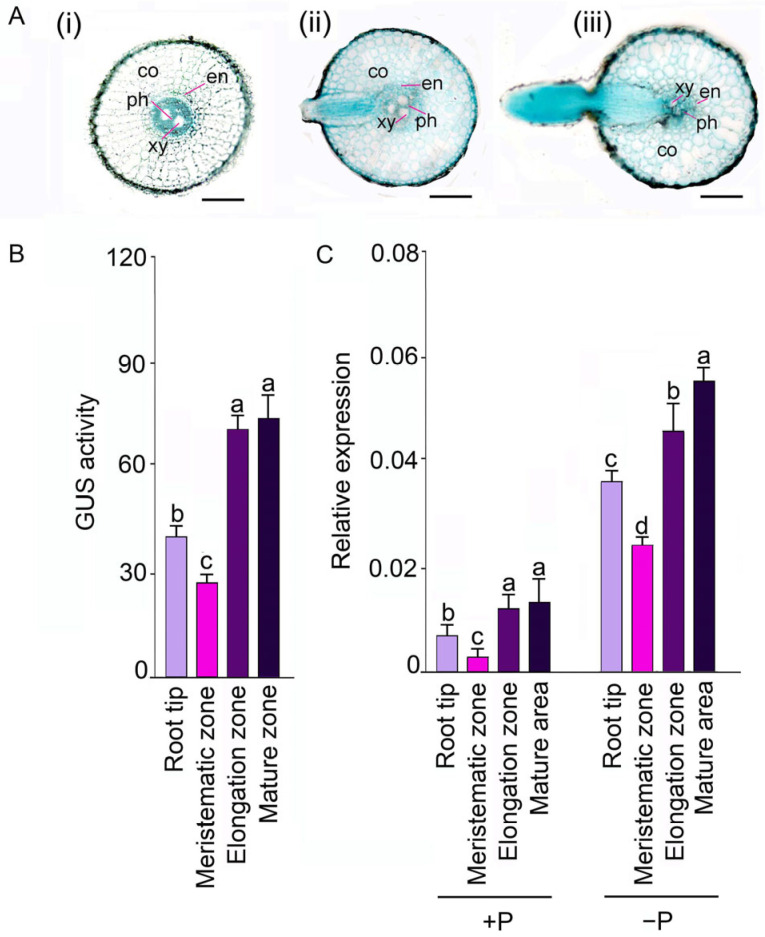
Expression pattern of *OsSQD1* in rice roots. Histochemical analysis of the tissue-specific GUS staining (**A**) and activity (**B**) driven by *OsSQD1* promoter (2028 bp upstream of its translation start codon). Cross-sections of *OsSQD1* promoter-GUS fusion roots in (**A**) were meristematic zone (i), elongation zone (ii), and mature zone (iii) from left to right. co, en, xy, and ph refers to cortex, endodermis, xylem, and phloem, respectively. Bars represent 100 μm. (**C**) The relative expression of *OsSQD1* in root tip, meristematic zone, elongation zone, and mature zone of roots under different +P and −P conditions. *Actin* (*OsRac1*; LOC_Os03g50885) was used as an internal control. Values (**B**,**C**) are means ± SE (*n* = 3) and different letters on the histograms indicate that the values differ significantly (*p* < 0.05, one-way analysis of variance).

To understand the role of *OsSQD1* in root development during the early growth stage, *OsSQD1*::GUS transgenic seedlings were grown under normal P conditions for 14 days. As shown in Figure 1A, the cross-section of the root revealed GUS expression in the xylem parenchyma, endodermis, phloem and cortex and in meristematic, elongation and mature zones. Deep GUS staining was also found in the lateral root primordium and lateral root (Figure 1A). We further examined the GUS activity in different parts of the root. The GUS activity of *OsSQD1* for all four zones in roots was in the order of mature and elongation zones> meristematic zone > root tip (Figure 1B). Then, the steady-state transcript levels of *OsSQD1* in different parts of roots under +P and −P conditions for another three days were measured by real-time qPCR (Figure 1C). Pi deficiency significantly induced the expression of *OsSQD1* in all four zones compared with that under +P conditions. In addition, the expression pattern of *OsSQD1* under −P supply was similar to the pattern under +P conditions, except for the relative expression in the mature and elongation zones. The relative expression of *OsSQD1* in the mature zone was significantly higher than that in the elongation zone (Figure 1C). The expression pattern of *OsSQD1* in roots revealed that *OsSQD1* may play an important role in the development of roots, especially lateral roots, which form and develop in the elongation zone and mature zone.

For further study, a complete homozygous *OsSQD1* knockout mutant (*ossqd1*) with a T-DNA insertion and 2 RNA interference (RNAi)-mediated knockdown lines were validated by quantitative RT-PCR and semiquantitative RT-PCR and used for root phenotype identification (Figure S1). The copy number of RNAi lines was detected by Southern blotting, and transgenic lines with a single copy were selected (Figure S1). The root phenotype of 14-d-old transgenic seedlings was observed under +P conditions (Figure S2). It was found that the knockout/down of *OsSQD1* inhibited the growth of primary and adventitious roots. In particular, adventitious roots were severely affected by the downregulation of *OsSQD1*, resulting in almost no adventitious roots growing in the *ossqd1* mutant (Figure S2A). Statistical analysis showed that the knockout/down of *OsSQD1* reduced 59.3%/41.3% primary root length and 90.7%/62.5% adventitious root length compared with those in the WT (Figure S2B,C). Meanwhile, there was no significant difference in the root/shoot ratio between the *OsSQD1* knockout/down lines and the WT (Figure S2D). To further study the effect of *OsSQD1* on lateral roots under different Pi regimes, 7-d-old WT and *OsSQD1* transgenic seedlings were transplanted under different Pi regimes for another 7 days. The lateral roots developing from primary roots were spread gently to reveal their architectural details (Figure S2E). There was a significant reduction in the lateral root density of the *ossqd1* mutant (59.6 and 69.9%) and RNAi lines (30.9 and 30.6%) under +P and −P conditions compared with the WT, respectively (Figure S2F). Lateral root length was also significantly reduced by the mutation and silencing of *OsSQD1* (Figure S2). These results suggested that the knockout/down of *OsSQD1* inhibited root growth under different Pi conditions, especially the formation and development of lateral roots under −P conditions.

### 2.2. Mutation of OsSQD1 Decreased the Concentrations of Phospholipids and Glycolipids under −P Condition

**Figure 2:**
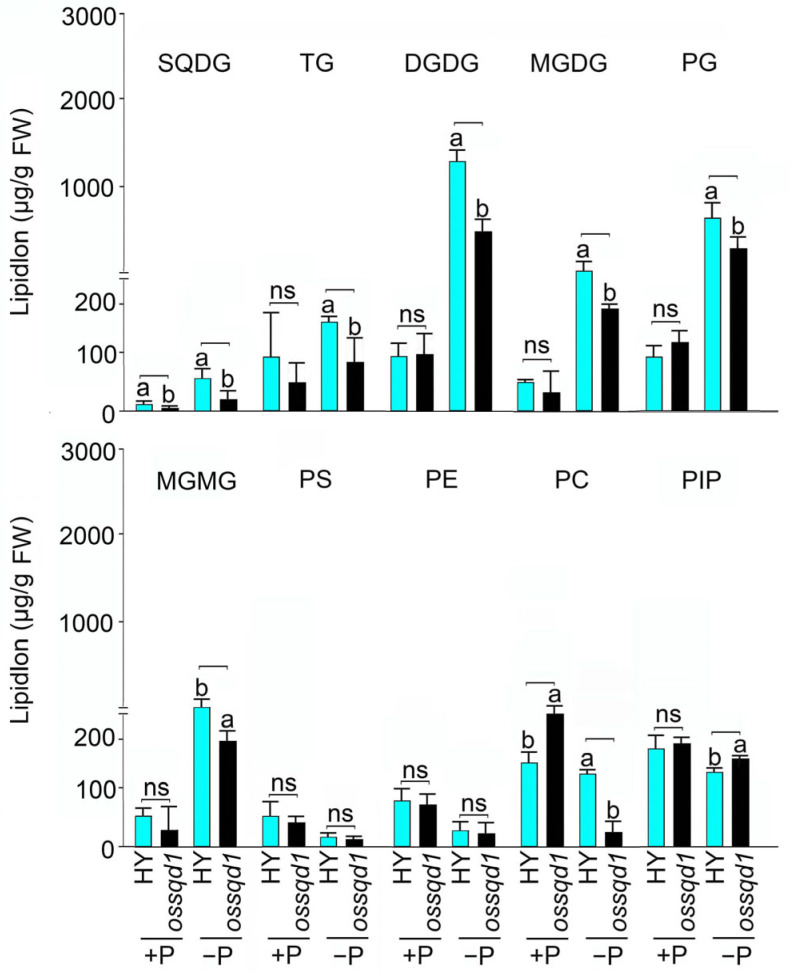
*OsSQD1* altered concentration of different lipid species in roots under different Pi regimes. The concentrations of different species of lipids in WT and *ossqd1* mutant roots under +P and −P conditions. SQDG, sulfoquinovosyldiacylglycerol; TG, triglyceride; DGDG, digalactosyldiacylglycerol; MGDG, monogalactosyldiacylglycerol; PG, phosphatidylglycerol; MGMG: monogalactosylmonoacylglycerol; PS, phosphatidylserine; PE, phosphatidylethanolamine; PC, phosphatidylcholine; PIP: phosphatidylinositol-4-monophosphate. Lipidlon replaces the concentration of lipid molecule. Values are means ± SE (*n* = 4) and different letters on the histograms indicate that the values differ significantly between WT and *ossqd1* mutant. (*p* < 0.05, one-way analysis of variance, Duncan’s test). ns = not significant.

The relative lipid intensity and lipid concentration of different lipid species in the *ossqd1* mutant shoots were exhibited in our previous study [23]. A description of the different lipid species’ concentrations of the *ossqd1* mutant roots under different Pi regimens is provided in this study. Under +P conditions, the mutation of *OsSQD1* markedly induced the concentration of PC compared with that in the WT. However, the concentrations of TG, DGDG, MGDG, PG, MGMG, PS, PE and PIP showed no significant change (Figure 2). Under Pi deficiency, the concentrations of different lipid species showed that PS and PE were not changed in the *ossqd1* mutant compared with WT rice. The analysis of TG (45.5%), DGDG (27.9%), MGDG (26.6%), PG (16%), MGMG (23%) and PC (78%) concentrations showed significant induction in *ossqd1* mutant roots. In addition, the concentration of PIP was slightly induced by *OsSQD1* mutation. The SQDG concentration was profoundly downregulated in *ossqd1* roots under both +P and −P conditions. These data suggested that the mutation of *OsSQD1* decreased the concentration of the majority of phospholipids and glycolipids under Pi deficiency.

### 2.3. Knockout/Down of OsSQD1 Changed the Composition of Different Lipid Species under Pi Deficiency

**Figure 3:**
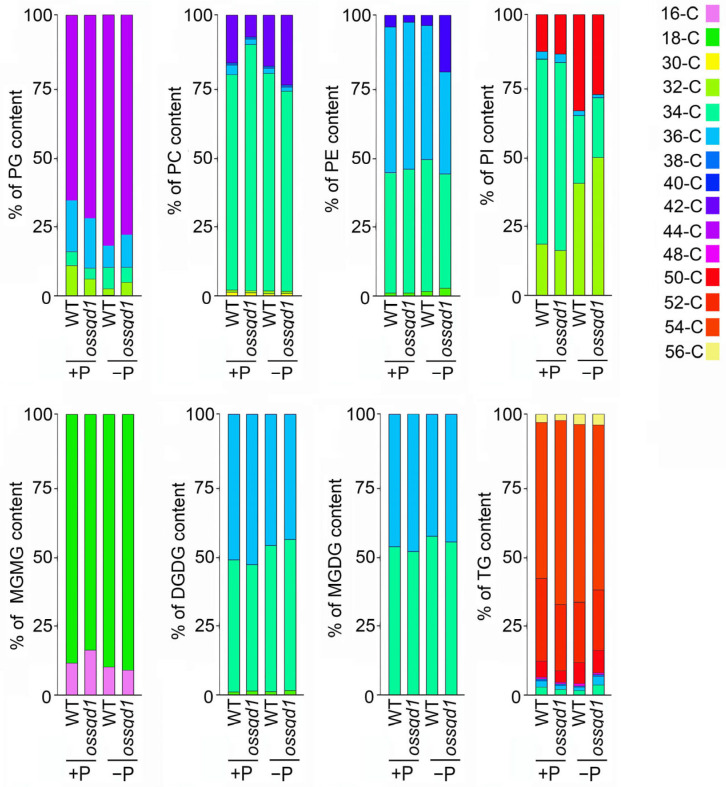
Knockout of *OsSQD1* changes the composition of phospholipids and glycolipids in roots under −P condition. Values show the proportion of the different acyl groups (groups of lipid species with the same number of acyl carbons) to the total class content, the sum of the normalized intensities of all the compounds belonging to the same class.

To understand the effect of *OsSQD1* on the composition of different lipid species, we examined the concentration of phospholipids and glycolipids with different acyl chain lengths under +P and −P conditions (Figure 3 and Figure S3). In contrast to the other phospholipids, the primary phospholipid in chloroplast inner membrane and thylakoid membrane is PG [48], which also showed the highest concentration in Pi-limited rice roots (Figure S3A). Mutation of *OsSQD1* had no effect on the concentrations of PGs with different acyl chain lengths in Pi-deficient roots. However, the 34-C, 36-C and 44-C PG concentrations were much higher in the *ossqd1* mutant roots than in the WT roots under −P conditions (Figure S3A). The effect of *OsSQD1* on PC composition was more complex. Under +P conditions, the 34-C and 36-C PC concentrations were increased in *ossqd1* mutant roots. The 34-C, 36-C and 42-C PC concentrations were decreased significantly in the *ossqd1* mutant compared with the WT under Pi deficiency (Figure S3B). In addition, the 34-C and 36-C PE concentrations were reduced by the mutation of *OsSQD1* under −P conditions (Figure S3C). The effect of *OsSQD1* on the changes in PI in response to Pi limitation was quite different from that on the changes in other phospholipids. *OsSQD1* mutation made the concentrations of 32-C and 34-C PI higher than those in WT roots under Pi deficiency (Figure S3D). There was no difference between the *ossqd1* mutant and WT in the concentration of glycolipids with different chain lengths. Interestingly, the mutation of *OsSQD1* under Pi deficiency had an impact on the glycolipid concentration of roots that could not be ignored (Figure S3E–G). Mutation of *OsSQD1* resulted in a decrease in 34-C/36-C DGDG and MGDG under Pi deficiency (Figure S3E,F). Furthermore, the concentrations of 32-C and 34-C SQDG were profoundly reduced under −P conditions (Figure S3G). TG species with long acyl chains were found in rice roots. The concentration of 52-C was increased by the *OsSQD1* mutation under Pi-sufficient conditions. Under −P conditions, the 50-C, 52-C and 54-C TG levels were considerably lower than those in the WT (Figure S3H).

**Figure 4:**
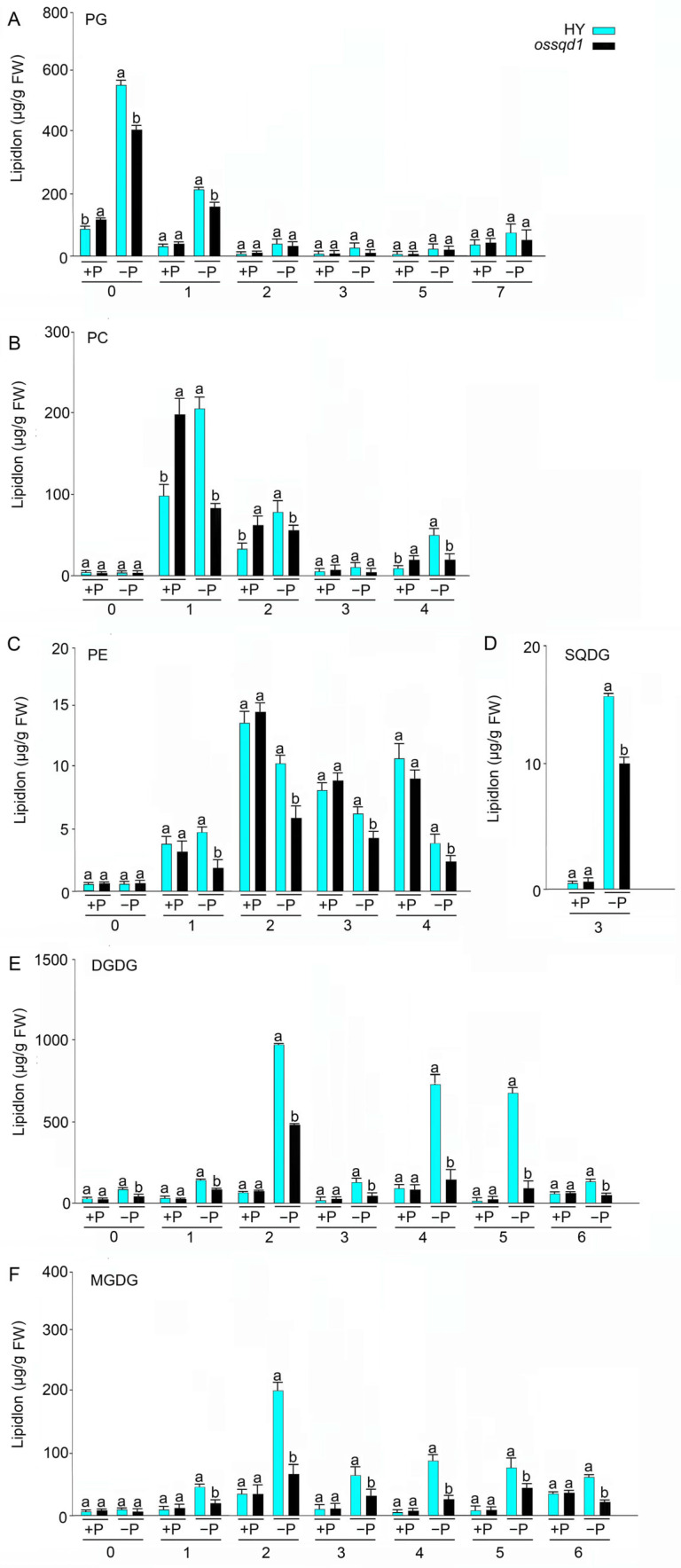
Knockout of *OsSQD1* affects the fatty acid saturation of different lipid species under different Pi regimes. The lipid saturation of PG (**A**), PC (**B**), PE (**C**), SQDG (**D**), DGDG (**E**) and MGDG (**F**). The abscissa represents the number of unsaturated bonds. Values are means ± SE (*n* = 4). Different letters indicate that the values differ significantly between WT and *ossqd1* mutant (*p* < 0.05, one-way analysis of variance, Duncan’s test).

To better visualize the changes in different lipid compositions, the proportion of different lipid species with various acyl chain lengths under +P and −P conditions in the *ossqd1* mutant is shown in Figure 4. Under Pi sufficiency, the proportion of 32-C PG was slightly decreased while 44-C PG was increased in *ossqd1* mutant roots compared with WT. Additionally, mutation of *OsSQD1* caused an increase in the 34-C PC proportions together with a significant decrease in 42-C PC. Nevertheless, the composition of phospholipids in *ossqd1* mutant roots was quite different from that in WT roots under Pi deficiency (Figure 3). The proportion of 36-C PG out of the total PG increased at the expense of 34-C PG in *ossqd1* mutant roots. There was a slight decrease in the 34-C PC together with the increased proportion of 42-C PC. For PE, the proportion of 36-C PE was strongly decreased, while that of 40-C PE was induced in *ossqd1* mutant roots under −P conditions. As observed for PG and PC, the proportion of 32-C PI was increased in Pi-deprived roots of the *ossqd1* mutant. Meanwhile, mutation of *OsSQD1* reduced the proportion of 50-C PI under −P conditions (Figure 3). Among glycolipids, the most obvious change in *ossqd1* mutant roots was observed for the composition of MGDG and DGDG under −P conditions. The proportion of 32-C MGDG and DGDG increased together with a slight decrease in 34-C MGDG and 36-C DGDG in Pi-deprived roots of the *ossqd1* mutant (Figure 3). In addition, the proportions of 34-C and 36-C TG were increased in the *ossqd1* mutant compared with WT (Figure 3).

### 2.4. Knockout/Down of OsSQD1 Decreased the Unsaturated Double Bonds of Glycolipids and Phospholipids under Pi Deficiency

Previous studies have indicated that fatty acid saturation is involved in abiotic stress tolerance [49]. The fatty acid saturation in roots of the WT and *ossqd1* mutant was employed and analyzed under different Pi regimes (Figure 4). Mutation of *OsSQD1* significantly increased the saturated fatty acid content of PG under +P conditions. Under Pi deficiency, the number of PG with zero and one unsaturated double bonds was significantly decreased in *ossqd1* roots (Figure 4A). Similarly, the number of fatty acids of PC with one, two, and four unsaturated double bonds increased, respectively, in *ossqd1* roots compared with WT roots under +P conditions. However, these fatty acids decreased significantly in number in the Pi-deficient roots of *ossqd1* (Figure 4B). No effect was observed in Pi-sufficient *ossqd1* roots for PE fatty acids, but the fatty acids with one, two, three, and four unsaturated double bonds decreased markedly in number upon the mutation of *OsSQD1* (Figure 4C). Due to the extremely low content of SQDG in roots under +P supply, the knockout of *OsSQD1* did not result in a significant decline in SQDG in roots (Figure 4D). By comparison, SQDG with three unsaturated double bonds was observed to be extremely low in number under −P conditions (Figure 4D). The fatty acid concentrations of DGDG and MGDG showed a similar trend. The fatty acids with one, two, three, four, five and six unsaturated bonds of DGDG and MGDG were all decreased significantly in *ossqd1* roots only under −P conditions (Figure 4E,F). These data indicated that the unsaturated double bonds of different lipid species were reduced by mutation of *OsSQD1* in Pi-deprived roots.

**Figure 5:**
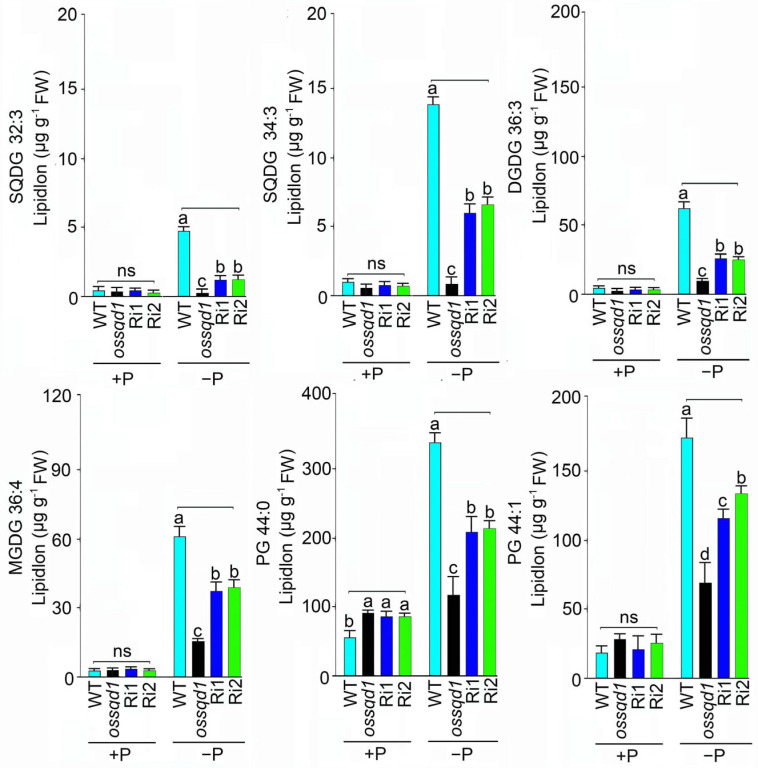
Knockout/down of *OsSQD1* decreased the fatty acid concentration of different phospholipids and glycolipids in roots under −P condition. Values are means ± SE (*n* = 4). Different letters indicate that the values differ significantly between WT, *ossqd1* mutant, Ri1 and Ri2 (*p* < 0.05, one-way analysis of variance, Duncan’s test). ns = not significant.

To explore the changes in fatty acid concentrations affected by *OsSQD1*, knockout/down transgenic lines (*ossqd1*, Ri1 and Ri2) were used to measure fatty acid concentrations under different Pi conditions. The fatty acid concentrations of glycolipids MGDG, DGDG and SQDG showed that the polyunsaturated fatty acids SQDG (C32:3), SQDG (C34:3), DGDG (C36:3) and MGDG (C36:4) were markedly decreased by the mutation of *OsSQD1* under −P conditions (Figure 5). As expected, all these fatty acids of glycolipids showed no change in *ossqd1* roots compared with WT roots under Pi-sufficient conditions (Figure 5). Regarding the fatty acid concentrations of PG, the concentrations of C44:1 in Pi-sufficient roots of *ossqd1* were similar to those in WT roots. Mutation of *OsSQD1* resulted in a significant increase in C44:0 PG under +P conditions (Figure 5). In contrast, the knockout/down of *OsSQD1* decreased C44:0 and C44:1 markedly compared with those in the WT (Figure 5). In summary, these results indicated that the mutation of *OsSQD1* triggered responses in the composition and saturation of phospholipids and glycolipids to Pi supply. The concentrations of long-chain unsaturated fatty acids, phospholipids and glycolipids were generally decreased by the knockout/down of *OsSQD1.*

### 2.5. Knockout/Down of OsSQD1 Affects the Lipid Remodelling Regulatory Network in Roots under −P Conditions

**Figure 6:**
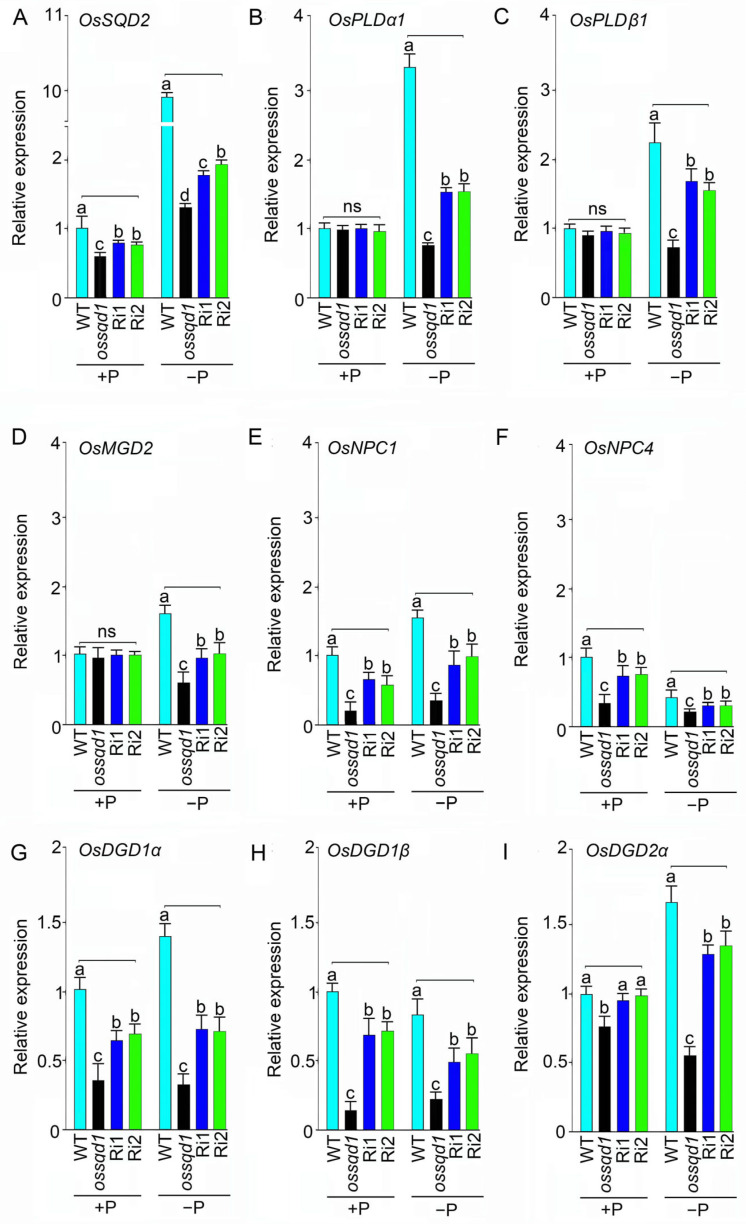
Knockout/down of *OsSQD1* affects the relative expression of lipid biosynthesis-related genes in roots under different Pi regimes. (**A**–**I**) Roots of WT, *ossqd1* mutant, Ri1 and Ri2 were harvested for assaying the relative expression of the different lipid biosynthesis-related genes (**A**–**I**, *OsSQD2.1*, *OsPLDα1*, *OsPLDβ1*, *OsPLDβ2*, *OsMGD2*, *OsDGD1α*, *OsNPC1*, *OsNPC4, OsDGD2α* and *OsDGD1β*) by qRT-PCR analysis. *OsACTIN* were used as internal controls. Values are means ± SE (*n* = 4). Different letters indicate that the values differ significantly between WT, *ossqd1* mutant, Ri1 and Ri2 (*p* < 0.05, one-way analysis of variance, Duncan’s test).

To investigate the effect of knockout/down of *OsSQD1* on the lipid remodeling regulatory network under +P and −P conditions, the expression of several genes related to lipid remodeling was measured by qRT-PCR in the *ossqd1* mutant and RNAi lines (Ri1 and Ri2). The genes analyzed were *OsSQD2.1* (LOC_Os07g01030), *OsMGD2* (LOC_Os02g55910), *OsDGD1β* (LOC_Os04g34000), *OsDGD1α* (LOC_Os02g33580), and *OsDGD2α* (LOC_Os03g11560), which are involved in glycolipid synthesis; *OsPLDβ1* (AK073012) and *OsPLDα1* (AK065102)*,* which are associated with phospholipid degradation; and *OsNPC1* (AK067741) and *OsNPC4* (AK243286), which function in hydrolyzing phospholipids and galactolipids (Figure 6). As shown in Figure 6, the relative expression of *OsPLDα1*, *OsPLDβ1* and *OsMGD2* was unaffected by the knockout/down of *OsSQD1* under +P conditions (Figure 6B–D). Nevertheless, the relative expression of *OsSQD2*, *OsNPC1*, *OsNPC4*, *OsDGD1α* and *OsDGD1β* in roots was reduced by knockout/down of *OsSQD1* under sufficient Pi (Figure 6A,E–H). The relative expression of *OsDGD2α* was downregulated only by the mutation of *OsSQD1* (Figure 6I). These results indicated that the effect of *OsSQD1* was much more obvious in Pi-deficient roots. The relative expression of all these genes was markedly decreased in the roots of the *ossqd1* and RNAi lines (Figure 6A–I). This result suggested that the knockout/down of *OsSQD1* caused the downregulation of phospholipid degradation and glycolipid synthesis genes under −P conditions.

## 3. Discussion

Lipids are the major components of the membranes of photosynthetic and nonphotosynthetic organs [6]. Pi-deprived plants utilize membrane phospholipids as an internal Pi source. To maintain membrane functionality, phospholipids are replaced by galactolipids [14]. Earlier, we demonstrated that *OsSQD1* encodes UDP-sulfoquinovose synthase, which is the enzyme catalyzing the first step of SQDG synthesis in rice. It plays an essential function in maintaining the homeostasis of sulfur and P [50]. Additionally, glycolipids were reduced by the mutation of *OsSQD1,* causing the inhibition of photosynthesis in Pi-deficient shoots [23]. In this study, we highlight the function of *OsSQD1* in the root phenotype under different Pi regimes (Figure S2). Knockout/down of *OsSQD1* significantly reduced the formation and development of lateral roots and the growth of primary and adventitious roots, especially under −Pi conditions [50] (Figure S2E,F). Mutation of *OsSQD1* significantly reduced PG, PC, SQDG, MGDG and DGDG concentrations under −P conditions (Figure 2). This finding highlights that the changes in different lipid species concentrations may have some effects on root development in response to external Pi supply. The overexpression of *LPA2* (*lysophosphatidic acid acyltransferase*) increases the content of major phospholipid classes and root length under phosphate starvation [51]. Additionally, *PPGP* (*phosphatidylglycerophosphate phosphatase*) can enhance primary and lateral root development in Pi-deprived Arabidopsis [52]. This result suggested that PG and PG biosynthesis was required for root growth and development in Arabidopsis under −Pi conditions. As *OsSQD1* encodes UDP-sulfoquinovose synthase, mutation of *OsSQD1* should hinder the synthesis of SQDG [50] (Figure 2). Therefore, the SQDG content may also have a certain impact on root growth and development. The influence of MGDG and DGDG on root development was not clear. However, it has been reported that an increase in the DGDG: MGDG ratio may alter membrane permeability and fluidity under abiotic stress [53]. These results suggested that the knockout/down of *OsSQD1* may inhibit root development by decreasing the contents of both phospholipids (PG) and glycolipids (SQDG, MGDG and DGDG) in Pi-deprived roots.

Mutation of *OsSQD1* altered the composition of PG, PC, PE, PI, DGDG, MGDG and TG in Pi-deprived roots (Figure 3 and Figure S3). SQD2 is the sulfoquinovosyltransferase catalyzing the final step in the SQDG biosynthetic pathway [43,54]. The *OsPLD* (phospholipase D) family encodes rice phospholipases, hydrolyzing phospholipids to generate PA and a free radical [55]. NPC4 (a nonspecific phospholipase C) produces DAG that is converted to PA, affecting the C18/C16 of DAG in Arabidopsis and playing a positive role in root growth [56]. OsMGD2 is the MGDG synthase (MGD) that is significant for the accumulation of MGDG [57]. It can transfer a galactosyl residue from UDP-Gal to diacylglycerol [58,59,60]. In Arabidopsis, the enzymes MGD2 and MGD3 preferentially synthesize 16:0 and 18:2 MGDG, while DGD2 preferentially uses 16:0 [38,39]. Our data showed that the knockout/down of *OsSQD1* downregulated the relative expression of *OsSQD2*, *OsPLD1α, OsPLD1β, OsMGD2, OsNPC4, OsDGD1α, OsDGD2α* and *OsDGD1β* upon Pi deprivation (Figure 6). This indicates that the lipid species’ composition alteration in *ossqd1* mutant roots during Pi deficiency may be the result of changes in enzymatic reactions.

Several environmental parameters, especially temperature, have a significant impact on the physical properties of membranes and affect the fluidity of the membrane. It is generally believed that maintaining the integrity and fluidity of membranes is essential for plants to survive under extreme environmental conditions [61]. The unsaturation level and acyl chain length of different lipid species affect membrane fluidity [62]. A high level of unsaturated double bonds in the main membrane lipids may contribute to greater tolerance to low temperatures [63]. In Pi-deprived roots of the *ossqd1* mutant, unsaturated double bonds were generally decreased in both phospholipids and glycolipids. This might affect the fluidity of the membrane, which may be a reason why the development of roots was inhibited more strongly in the *ossqd1* mutant under −P conditions.

Compared with our previous research, this research revealed an obvious difference between the effect of *OsSQD1* on the lipid concentration and composition of roots and shoots [23] (Figure 2, Figure 3, Figure 4 and Figure 5). In Pi-deprived shoots, the concentrations of PG, LPG, TG, and PC were induced by mutation of *OsSQD1*. Nevertheless, the concentrations of TG, PG and PC were greatly reduced in Pi-deprived roots of the *ossqd1* mutant [23] (Figure 2 and Figure S3). These changes were accompanied by increases in 32-C PG, 34-C PG, 34-C PC and 36-PC in shoots under −P conditions. The concentrations of 32-C and 34-C PG and 34-C and 36-C PC were significantly decreased in roots upon Pi deficiency and were affected by the mutation of *OsSQD1* [23] (Figure 3 and Figure S3). The glycolipid changes showed that 36-C MGDG, 34-C and 36-C DGDG were significantly reduced in Pi-deprived shoots of the *ossqd1* mutant. These data were also slightly different from those on Pi-deprived roots [23] (Figure S3). According to previous studies, the concentration and composition of different lipid species in plant roots under conditions responding to Pi deficiency were different from those in shoots. Under −P conditions, the concentrations of PC, PE, PG, and PS were significantly lower than those under Pi sufficiency. However, the concentrations of PC and PS did not change, and PG was highly induced by Pi deficiency in both rice and Arabidopsis roots [20,23] (Figure 2). These results indicated that there may be distinctions in lipid metabolism in response to Pi deficiency between roots and shoots in rice, which caused the distinctions in the role of *OsSQD1* in overall lipid homeostasis between roots and shoots.

## 4. Materials and Methods

### 4.1. Creation and Acquisition of Plant Materials

The *ossqd1* mutant (PFG_2B-30,198.R) with T-DNA insertion in wild-type (WT; *Oryza sativa* L. ssp japonica ‘Hwayoung’) background was acquired from the rice T-DNA insertion mutant database RiceGE (http://signal.salk.edu/cgi-bin/RiceGE, accessed on 11 December 2022). The RNAi lines (Ri1 and Ri2) and *OsSQD1* promoter-GUS fusion line were created as described in Sun et al., 2020. The primers used for confirming the insertion of T-DNA, and the transcript abundance of *OsSQD1* were listed in Table S1. The promoter region of *OsSQD1* was amplified from the genomic DNA using gene-specific primers with restriction sites *Kpn*I and *Hind*III (Table S1).

### 4.2. Plant Growth and Treatment Conditions

Seeds of WT, *ossqd1* mutant, Ri1 and Ri2 lines were germinated in the dark at 37 °C. WT and transgenic lines seeds were grown hydroponically for 10 d in the normal Pi solution making up with MgSO_4_ (1 mM), NH_4_NO_3_ (1.25 mM), Na_2_SiO_3_ (0.5 mM), KH_2_PO_4_ (0.2 mM), CaCl_2_ (1 mM), EDTA-Fe (20 µM), K_2_SO_4_ (0.35 mM), H_3_BO_3_ (20 µM), ZnSO_4_ (0.77 µM), (NH_4_)_6_Mo_7_O_24_ (0.39 µM), MnCl_2_ (9 µM), and CuSO_4_ (0.32 µM) with pH adjusted to 5.5. The 10-d-old seedlings were then transferred to the +P or –P (10 µM Pi) solutions for another 7 d (analysis of lipid-remodeling regulatory network) or 14 d (analysis of lipid and fatty acid concentration) as required. Rice was grown under controlled conditions in a light incubator (16 h light, 30 °C/8 h dark, 22 °C, relative humidity ~70%).

### 4.3. GUS Assays

Histochemical localization of GUS activity was detected by incubating 14-d-old roots with the substrate X-gluc (Research Organics, Cleveland, OH, USA), as described by Sun et al., 2020 [50]. Fluorometric assay of the bacterial β-glucuronidase (GUS) activity was detected as described in [64].

### 4.4. RNA Extraction and Expression Analyses

Total RNA (~1 μg) was extracted from roots of WT, *ossqd1* mutant, Ri1 and Ri2 using TRIzol reagent (Invitrogen) for the synthesis of cDNA. First-strand cDNAs were synthesized from total RNA using the Goldenstar™ RT6 cDNA Synthesis Kit (TSINGKE, Shanghai, China). qRT-PCR was performed on LightCycler 480 II (Roche, Switzerland) using SYBR Green qPCR Mix (Tsingke, China) with 3 biological replicates, each with 3 technical repeats. The cycling conditions were as follows: 95 °C for 30 s, 35 cycles of 95 °C for 5 s and 60 °C for 30 s. Gene-specific primers used for qRT-PCR are listed in Table S1. *ACTIN* (*OsRac1;* LOC_Os03g50885) was used to normalize the reaction. Relative expression of the genes was computed by the 2 ^–ΔΔCT^ method as described in Livak and Schmittgen, 2001 [65].

### 4.5. Quantification of Different Lipid Species and Fatty Acids

The roots of WT, *ossqd1* mutant, and RNAi lines grown in +P and −P conditions for 21 d were homogenized into powder in liquid nitrogen. 200 μL cold water, 20 μL lipid internal standard mixture and 800 μL cold methyl tert-butyl ether were added to the samples then vortexed for 30 s. Following this, 240 μL methanol was added to the samples and adequately vortexed, sonicated at 4 °C for 20 min and left to stand for 30 min, then centrifuged (14,000× *g* for 15 min at 10 °C) to extract lipids. The upper organic layer was dried in a vacuum centrifuge. The lipid extraction, seperation and quantification was detected as described in Sun et al., 2021. The Lipidsearch 4.0 software was used for peak detection and annotation of lipids or internal standards.

### 4.6. Statistical Analysis

Data were analyzed by ANOVA using SPSS 20 program (www.spss.com, accessed on 11 December 2022). Duncan’s multiple range test at *p* < 0.05 was carried out for all the experiments to determine the significance between the WT and knockout/down lines.

## 5. Conclusions

In this study, the function of *OsSQD1* in root development and growth under different Pi regimes was identified. Knockout/down of *OsSQD1* obviously inhibited lateral root formation and development under −P conditions. The analysis of the concentration and composition of different lipid species in Pi-deprived roots of the *ossqd1* mutant showed that the mutation of *OsSQD1* decreased the concentration and the unsaturated fatty acids of phospholipids and glycolipids in roots under −P conditions. Additionally, it also decreased the concentration of fatty acids with different acyl chain lengths, altering the composition of lipids under Pi deficiency. These results indicate that *OsSQD1* influences root development under −P conditions by affecting lipid composition and saturation in rice.

## Data Availability

Not applicable.

## Supplementary Materials

The following supporting information can be downloaded at: https://www.mdpi.com/article/10.3390/ijms24010114/s1.
