# Supplementary material for: Does Sulfoquinovosyl Diacylglycerol Synthase OsSQD1 Affect the Composition of Lipids in Rice Phosphate-Deprived Root?
Source: Int J Mol Sci. 2022 Dec 21;24(1):114. doi: 10.3390/ijms24010114 (PMC9820689; doi:10.3390/ijms24010114)
Supplement: Supplementary file 1 [file ijms-24-00114-s001.zip › ijms-2049497-supplementary.pdf]

Table S1.Primers used for the identification of *ossqd1* mutant and the construction of promoter-GUS and RNAi vectors.

S.No.	Purpose	Primer name	Primer sequence
1	Identification of *ossqd1* mutant	*OsActin*-RT-FP	GGAACTGGTATGGTCAAGG
		OsActin-RT-RP	AGTCTCATGGATACCCGCAG
		P1	TGCTGTTGCTGCTATCCATC
		P2	CTCACACCCATAGCCTCCAT
		LP	TCACCAAAGGTTAGCAGCAAC
		RP	TCTCTCAATTTTGGCCAAGC
		BP	ACGTCCGCAATGTGTTATTAA
		OsSQD1-GFP-RP	ATGGATCCTGGTAGAACTGTCAGT
2	Generation of RNAi knockdown lines for *OsSQD1*	*OsSQD1*-RNAi-FP1	ATGGTACCACTGTAGTTTCAGCCCTTCG
		OsSQD1-RNAi-RP1	TAGGATCCTGGCAACCTCGTATCCTT
		OsSQD1-RNAi-FP2	GGACTAGTACTGTAGTTTCAGCCCTTCG
		OsSQD1-RNAi-RP2	CGAGCTCTGGCAACCTCGTATCCTT
3	Generation of *OsSQD1* promoter-GUS reporter transgenic lines	*OsSQD1*-Promoter-FP	ATAAGCTTGGGTGATTGTGAGTGAGT
		OsSQD1-Promoter-RP	ATGGTACCCAGGCGAAGGGCTGAAAC

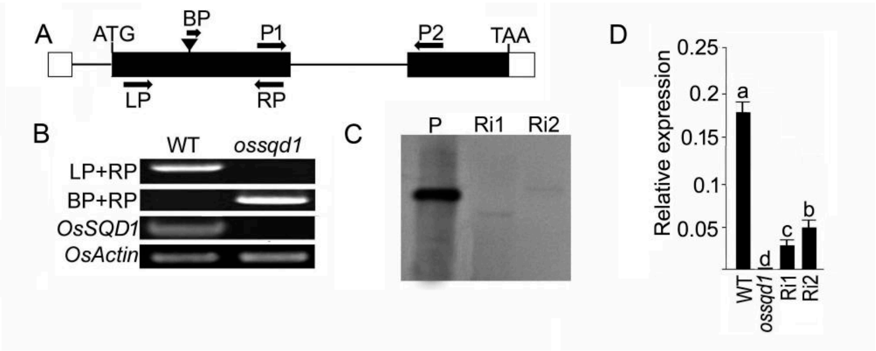

Figure S1. The isolation and validation of *OsSQD1* knockout/down mutants. (A) Schematic representation of the genomic organization of OsSQD1 showing distribution of the UTR (white boxes), exons (black boxes), intron (black line), the T-DNA insert in the exon (black triangle), and the location of the primer sets (BP, LP, RP, P1, and P2) used for identifying homozygous *ossqd1* mutant. (B) Semi-quantitative RT-PCR analysis was employed for determining the insertion of the T-DNA in the knockout mutant *ossqd1* by using primers of LP + RP, and BP + RP, and compared with the WT. (C) Southern blot analysis was performed for the detection of the *OsSQD1* copy number in independently generated RNAi lines (Ri1 and Ri2). Positive control (P) was generated by transforming the transgenic with an empty vector pTCK303. (D) The relative expression level of *OsSQD1* in *ossqd1* mutant, Ri1, and Ri2 were assayed by qRT-PCR analysis. *Actin* was used as an internal control. Values are means ± SE (n = 4) and different letters on the histograms indicate that the values differ significantly ($P < 0.05$, one-way ANOVA).

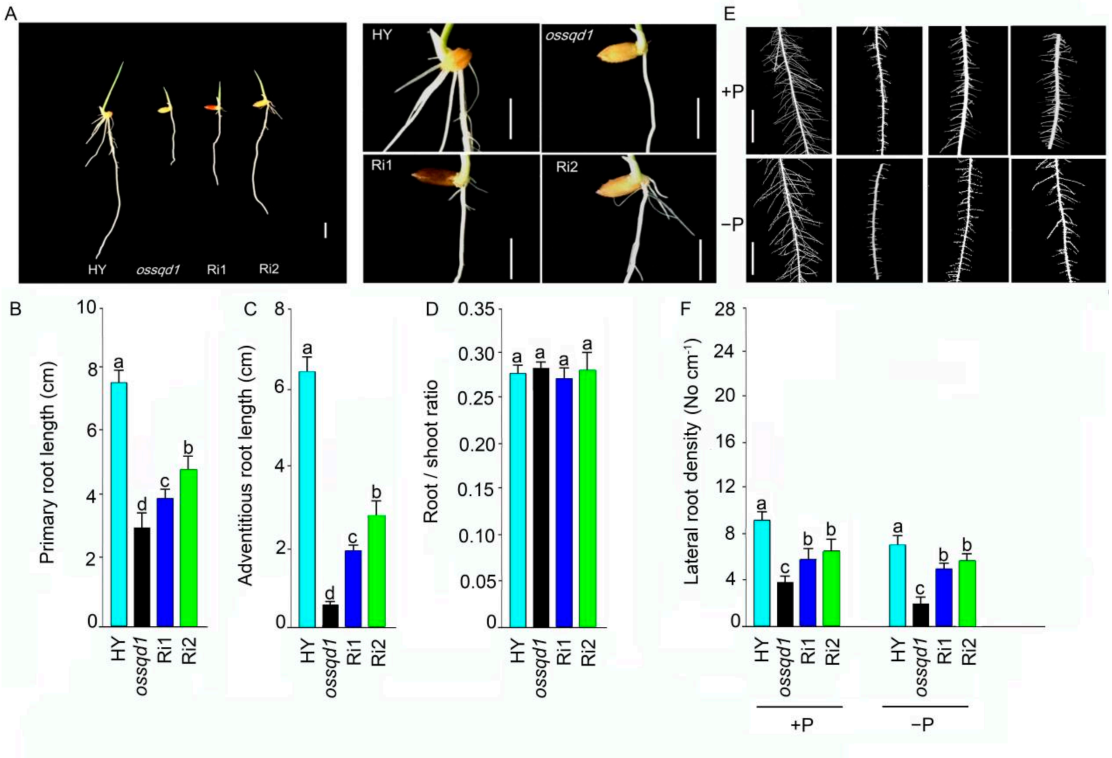

Figure S2. Knockout/down of *OsSQD1* affects the root development under different Pi regimes. (A) The phenotype of primary and adventitious roots of different transgenic lines under normal condition. (B-D) The primary root length (B), adventitious roots length (C) and root / shoot ratio (D) of different transgenic lines under normal condition. (E and F) The performance of lateral roots (E) and lateral root density (F) of different transgenic lines under +P and -P conditions. Bars represent 0.5 cm (A) and 0.2 cm (E), respectively. Values (B-D and F) are means ± SE (n = 8) and different letters on the histograms indicate that the values differ significantly ($P < 0.05$, one-way analysis of variance, Duncan's test)

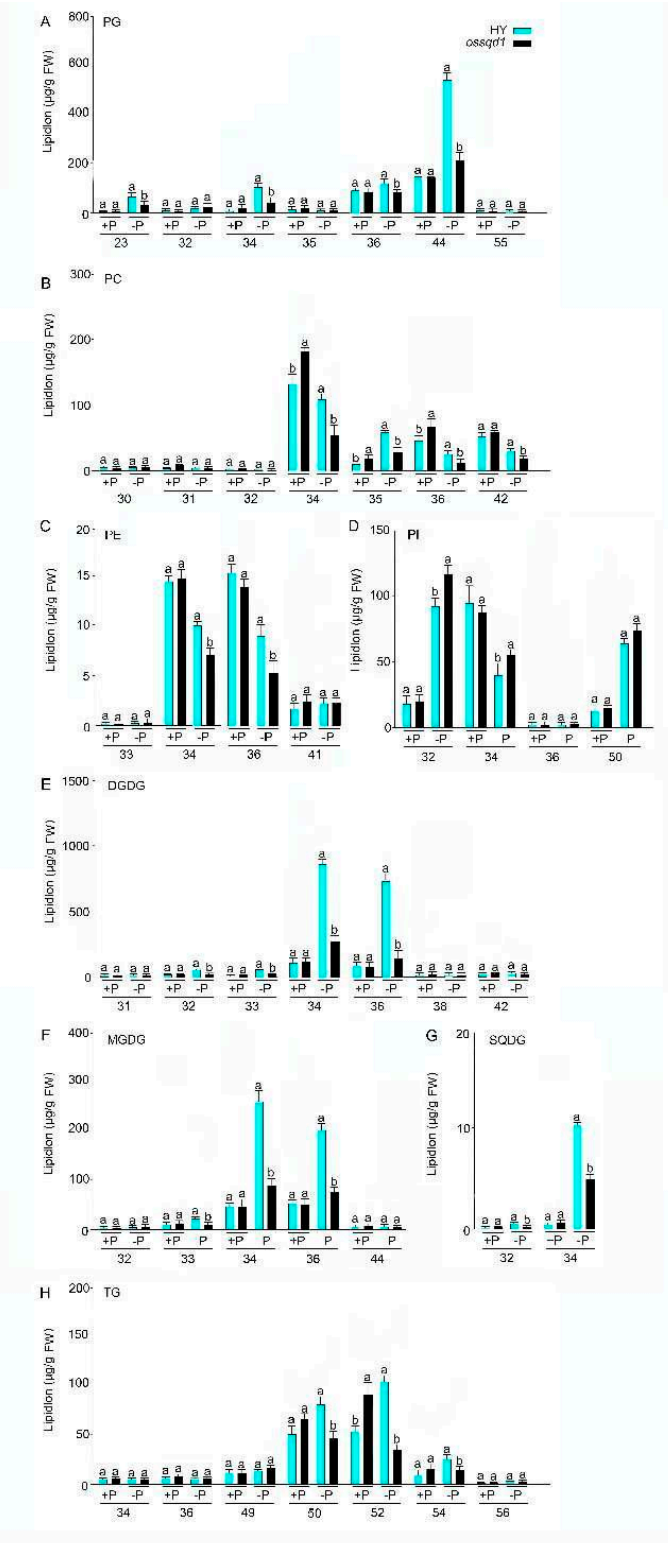

Figure S3. Knockout of *OsSQD1* changes the lipid composition of different species with different acyl chain length mainly under -P condition. (A-H) The concentration of lipid composition of PG (A), PC (B), PE (C), PI (D), DGDG (E), MGDG (F), SQDG (G), and TG (H). The abscissa represents lipid molecules with different acyl

chain lengths. Values are means ±SE (*n* = 4). Different letters indicates that the values differ significantly between WT and *ossqd1* mutant ($P < 0.05$, one-way analysis of variance, Duncan’s test).
